# The impact of the number of high temporal resolution water meters on the determinism of water consumption in a district metered area

**DOI:** 10.1038/s41598-023-46086-z

**Published:** 2023-11-02

**Authors:** Justyna Stańczyk, Krzysztof Pałczyński, Paulina Dzimińska, Damian Ledziński, Tomasz Andrysiak, Paweł Licznar

**Affiliations:** 1 Institute of Environmental Engineering, Wroclaw University of Environmental and Life Sciences, Grunwaldzki Sq. 24, 50-363 Wroclaw, Poland; 2 Faculty of Telecommunications, Bydgoszcz University of Science and Technology, Computer Science and Electrical Engineering, Profesora Sylwestra Kaliskiego 7 St, 85-796 Bydgoszcz, Poland; 3 MWiK—The Water Supply and Sewerage Company of Bydgoszcz Sp. zo.o., 103 Toruńska St., 85-817, Bydgoszcz, Poland; 4 Faculty of Building Services, Hydro and Environmental Engineering, Warsaw University of Technology, Nowowiejska St. 20, 00-653 Warszawa, Poland

**Keywords:** Environmental sciences, Engineering, Mathematics and computing

## Abstract

Developments in data mining techniques have significantly influenced the progress of Intelligent Water Systems (IWSs). Learning about the hydraulic conditions enables the development of increasingly reliable predictive models of water consumption. The non-stationary, non-linear, and inherent stochasticity of water consumption data at the level of a single water meter means that the characteristics of its determinism remain impossible to observe and their burden of randomness creates interpretive difficulties. A deterministic model of water consumption was developed based on data from high temporal resolution water meters. Seven machine learning algorithms were used and compared to build predictive models. In addition, an attempt was made to estimate how many water meters data are needed for the model to bear the hallmarks of determinism. The most accurate model was obtained using Support Vector Regression (8.9%) and the determinism of the model was achieved using time series from eleven water meters of multi-family buildings.

## Introduction

Intelligent Water Systems (IWSs) are one of the components of smart cities, an idea that is constantly being implemented and refined as part of the vision of the city of the future. The intelligence of the water sector, according to the Water Environment Federation, is evidenced by the use of advanced technologies in decision-making and management^1^. In practice, this means, among other things, the need to reduce operational costs, manage and mitigate risks, which should be facilitated by risk assessment solutions, failure prediction, performance prediction and decision support systems^2,3^. In the context of water supply networks, intelligence is sought in Automatic Meter Reading (AMR) and machine learning methods for analyzing and interpreting recorded time series, preferably in real time^4^.

Water Distribution Network (WDN) operation and optimization are based on the division of the water supply network into District Metered Areas (DMAs). With the help of control and measuring devices, isolating and cutting-off valves and Pressure Reducing Valves (PRVs), water supply network operators are able to balance the volume of water pumped into a specific zone, manage pressure and reduce water losses due to leaks^5^. The optimal number of nodes forming a water supply zone should be in the range of 500 to 5,000^6^, as overextended zones will cause the amount of non-revenue water to increase. On the contrary, if the number of nodes is too small, implementing monitoring of the water supply network can be costly. Therefore, network managers face the problem of selecting the optimal number of measuring devices so as to acquire as much diagnostically useful information as possible with the least number of them. Smart metering sensors installed in DMAs contribute to water, energy and economic savings, which, according to Spedaletti et al.^7^, translates into financial savings, gained from locating and fixing uncontrolled leaks, of €1,857 over 3 months for the city of Osimo (Italy), with a population of more than 33,000. From the level of the water utility user, installing smart meters and allowing consumers to view their current water consumption additionally allows water savings in the range of 15–26%^8^, which significantly supports the push for sustainable cities.

Determining the boundaries of DMA zones, selecting the optimal number of nodes or locating the installation of PRVs are the scope of research using graph theory, clustering analysis, multi-objective optimization and multi-criteria analysis^9,10^. The basis for clustering includes such data as the amount of water demand, the topography of the area, and the coordinates of the line axes at contractual nodal points. Apart from the issues concerning the delineation of DMA boundaries, it is also important to try to answer questions about how to deploy measuring devices and their number so that monitoring data can be used effectively by decision makers. On the one hand, a large amount of measuring devices makes it possible to visualize detailed conditions of the hydraulic flow of water in pipes, but on the other hand, it can create streams of data that are impossible to process in the context of network operating condition detection^11^. In addition, each measuring device generates a certain cost of its purchase and subsequent operation, so measuring points should bring maximum benefit in the form of information useful to network operators. Brentan et al.^12^ proposed modifying the k-means algorithm for partitioning and using a multi-objective Particle Swarm Optimization (PSO) to suitably place partitioning devices. The results showed that this approach contributed to pressure control, reduced and faster leak detection, lowered the energy intensity of the system and increased its reliability.

The random nature of water consumption by users of water supply networks, hourly, daily and seasonal variability (as noted by Luna and Ballini^13^), cause the recorded time series of hydraulic water flow to be treated as a stochastic process. The non-stationary, non-linear, and inherent stochasticity of water consumption data at the level of a single water meter means that the characteristics of its determinism remain virtually impossible to observe^14^. Additionally, overly rigorous data pre-processing can lead to the omission of dynamic behaviour in water consumption^15^. Rahim et al.^16^, showed that 15-min profiling is the most appropriate interval for clustering based on consumer behaviour similarity. Learning about water consumers' habits, certain routines and customs is an important issue in creating water demand strategies^17^. As Rahim et al.^18^ noted in their study, sociodemographic data are crucial, but certain water consumption habits can only be learned when analyzing data at the water meter level. Even within the data recorded separately for each floor of a multi-family building, it was observed that water consumption reading was fluctuating, which is somewhat of an analytical barrier^14^. A study by Ramulongo et al.^19^ found that residents use the most water for cooking and bathing, although this is further influenced by factors such as the age of the residents, their education level, income level, the equipment of the apartments with water-consuming devices, the size of the building and its age^20,21^. Thus, water consumption by network customers shows quantitative changes over time, but also in the space operated by a given water supply system^22^. Predictive models using methods such as Random Forest (RF), Artificial Neural Networks (ANNs) or Support Vector Machines (SVR)^23–25^ attempt to forecast water consumption, an aspect already sufficiently explored by the scientific community.

This article hypothesizes that the exploration of measurement data from water meters should provide insight into the limit of the visibility of determinism in the time series of water consumption within DMAs zones. Improper location of metering devices results in financial as well as time losses, and the data will not be effectively used in the context of assessing the operational status of the water supply network. The location of master devices—zonal flow meters, among others – which are later used to balance water in relation to data recorded by water meters, should allow for increasing the efficiency of water distribution systems^10^. While metering devices installed within DMAs enable data recording with a small time interval, as noted by Hu et al.^17^ there is still the problem of lack of high resolution data for water meter data. In addition, water consumption information is often collected from periodical readings in a non-simultaneous, cumulative form, which results in the accumulation of daily water consumption values^26^, making it difficult to predict water consumption at the consumer level. A significant number of water utilities use radio reading of water consumption data, which prevents ongoing water balancing based on zonal devices providing information with an interval of typically 10–15 min. The problem of not having smart metering with high resolution water consumption and not considering consumption data is a barrier to creating reliable forecasting models and water demand patterns that can later feed hydraulic models of water supply networks^7,18^.

In light of the existing limitations of water consumption analysis at the level of water meters with high temporal resolution and the lack of synchronized water meter data with high resolution with respect to DMA, an attempt was made to explore the data in the context of the possibility of determining the threshold beyond which water consumption bears the hallmarks of a deterministic model. The goal is to estimate from how many facilities of the type of multi-family building should water meter data be provided, so that during the short-term prediction of water demand one can talk about the determinism of the data and at the same time obtain a reliable prediction. The realization of the research assumptions was made using machine learning algorithms. The obtained research results can contribute to the optimization of the number and location of master measuring devices over water meters. The methodology for processing water meter data, developed and demonstrated in the article can support the process of calibrating hydraulic models by creating water demand patterns for several buildings or neighbourhoods simultaneously, especially in the absence of current water consumption data with high resolution at the level of water meters of individual facilities. The research also casts new light on the procedure for creating further sub-zones within a single DMA, for which reason it can be used as part of water demand management strategies created by decision makers.

## Materials and methods

### Forecasting process steps

The time series of consumer water consumption is a stochastic process exhibiting a deterministic and a random component. In the case of the deterministic component, it is possible to forecast its value using harmonic analysis, models of the Autoregressive Integrated Moving Average (ARIMA) or ANNs class. The value of the random component is probabilistic and can be determined by analyzing the results with an assumed probability of occurrence. Time series of water consumption represent discrete data that are subject to errors due to imperfections in the methods of measurement used, conversion of analogy data into digital data, transmission and archiving. These errors are called measurement noise. The mathematical notation of the stochastic water consumption process can be represented by Eq. (1) ^27^:

1 $$X_{t} = Z_{t} + S_{t} + \varepsilon_{t}$$

where: ${X}_{t}-$ recorded values of the time series, ${Z}_{t}-$ deterministic component, the fundamental movement of the series, ${S}_{t}-$ seasonality (periodic phenomenon), ${\varepsilon }_{t}-$ error or residual component, random part of the time series.

**Figure 1:**
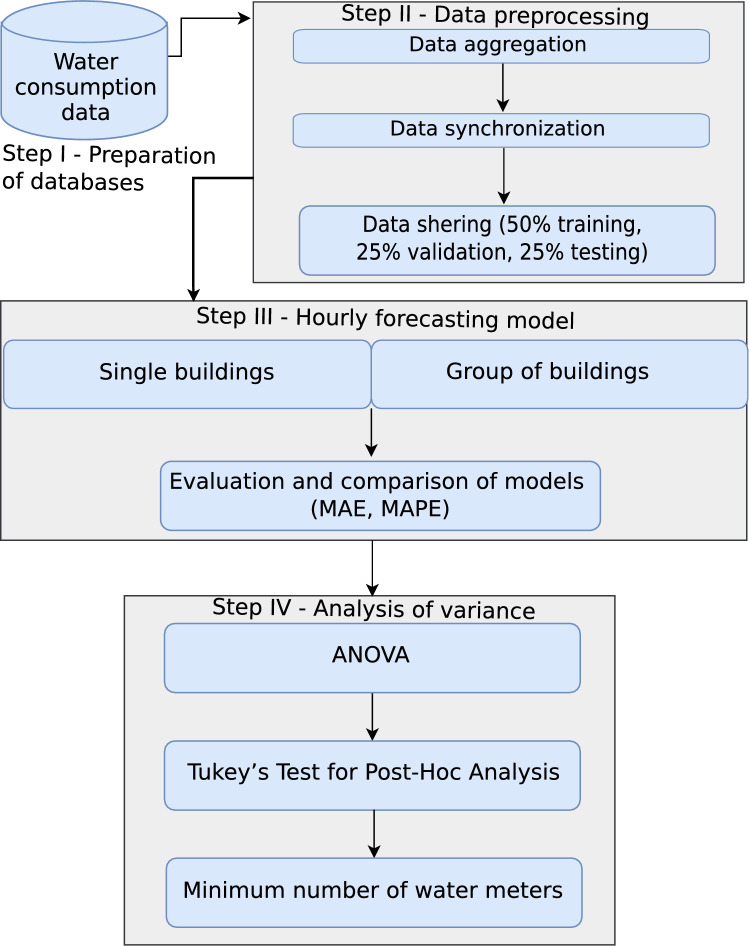
The methodology flowchart.

The research methodology was divided into different stages, shown in the methodology flowchart (Fig. 1). In the first step, water consumption data was collected from individual water meters installed in buildings with diverse purposes. In subsequent steps, data pre-processing was carried out, which involved aggregating the data to hourly water consumption and synchronizing them. Next, water consumption prediction was made and the accuracy of the predictions was evaluated. The final step involved analysis of variance of prediction errors via analysis of variance (ANOVA), which was used to determine the minimum number of water meters for which the distribution of prediction error is no longer distinguishable from the variant of water consumption forecasting based on the maximum number of water meters.

### Datasets preparation

The study used water consumption recorded with high resolution by water meters of buildings, in the area of the DMA zone, constituting the case study The water meter derived raw data was encoded as a series of time elapsed between flows of one liter of water. Crude water meter files were transformed by hourly grouping and summation. Finally, the time series of aggregated hourly water consumption was used for analysis.

Water consumption was projected over an hourly time horizon separately for a single residential block and a suitably sized group of blocks, starting from a pair of blocks up to nineteen blocks at a time. Each measurement week was assigned a corresponding index number and 50% of them constituted a learning set, 25% a validation set and 25% a test set. The algorithm used in the study randomly shuffled each week and building group and divided the data into individual sets, repeating the experiment thirty times.

### Machine learning methods

Prediction of hourly water consumption was made using Random Forest (RF), Fully-Connected Neural Networks (FCNN), Recurrent Neural Networks (RNN), XGBoost, Decision Tree, K-Nearest Neighbours (KNN) and Support Vector Regression (SVR) machine learning algorithms. Although various types of the above-mentioned algorithms and their hybrids are used in the scientific literature in water consumption prediction studies, each of them has its specific advantages, disadvantages and scope of application. In general, all of the above algorithms are used due to their inclusion of all major methods for extracting patterns from data. Neural Networks and Support Vector Regressors transform the input data to create a continuous function that returns a predicted value. The K-Nearest Neighbours algorithm clusters the search space to perform proximity-based regression. Decision Trees partitions the solution space into semi-clustered subplanes based on the purity of the partition. Random Forest and XGBoost, on the other hand, are ensemble-class meta-algorithms that combine Random Forest classifiers to create a more robust predictive model. All the necessary code for the described numerical experiments was developed in Phytone using dedicated libraries such as sklearn, pytorch, xgboost and Numpy.

### Evaluating forecast accuracy

The evaluation of the accuracy of the prediction of water distribution one hour ahead was based on Mean Absolute Error (MAE) and Mean Absolute Percentage Error (MAPE), along with their modifications to maximum and over mean values. A rather important issue from a methodological point of view is the impossibility of explicitly comparing the results obtained with other studies conducted in this area. The most commonly used measure for evaluating forecast accuracy is the MAPE error ^28–30^ or directly the RMSE ^24^. By default, MAE and MAPE error are determined via Eqs. (2) below (3):

2 $$MAE = \frac{{\mathop \sum \nolimits_{i = 1}^{n} \left| {y_{t} - \hat{y}_{t} } \right|}}{n}$$

3 $$MAPE = \frac{1}{n}\sum\nolimits_{t = 1}^{n} {\frac{{\left| {y_{t} - \hat{y}_{t} } \right|}}{n}} \cdot 100\%$$

where: $n-$ number of observations, ${\widehat{y}}_{t}-$ the value predicted by the model for time point $t$, ${y}_{t}-$ the value observed at time point $t$.

The authors of the research did not decide to use other metrics, e.g. Root Mean Squared Error (RMSE) due to the specificity of the studied phenomenon and its high dynamics which can be observed in water consumption time series. Therefore, a metrics was sought that would not be highly sensitive to outliers and the scale of the dependent variable, which are necessary in order to match the nature of the studied phenomenon and implement machine learning. As a more reliable metric for evaluating predictive models relative to classic MAE and MAPE errors, according to the authors, the MAE over mean was used (${MAE}_{oM}$) error described by Eq. (4):

4 $$MAE_{oM} = \frac{MAE}{{\frac{1}{n}\sum\nolimits_{i = 1}^{n} {y_{t} } }} = \frac{{\sum\nolimits_{i = 1}^{n} {\left| {y_{t} - \hat{y}_{{tc^{ - } }} } \right|} }}{{\sum\nolimits_{i = 1}^{n} {y_{t} } }}$$

The reason for this function application for metrics evaluation lies in the numerical properties of the examined signals. The MAPE error is not applicable due to the presence of zero-valued samples in the observed signal. Because $\underset{y\to {0}^{+}}{lim}\frac{|x-y|}{y}=\left|x\right|\underset{y\to {0}^{+}}{lim}\frac{1}{y}=\infty$ for every finite, non-zero $x$, the MAPE error for the signal with at least one sample equal to zero is equal to $MAPE =\frac{1}{n}{\sum }_{t=1}^{n}\frac{\left|{\widehat{y}}_{t}-{y}_{t}\right|}{{y}_{t}}=\frac{1}{n}\left\{{\sum }_{t=1}^{n-1}\frac{\left|{\widehat{y}}_{t}-{y}_{t}\right|}{{y}_{t}}+\underset{{y}_{n}\to {0}^{+}}{lim}\frac{|{x}_{n}-{y}_{n}|}{{y}_{n}}\right\}=\frac{1}{n}\left\{{\sum }_{t=1}^{n-1}\frac{\left|{\widehat{y}}_{t}-{y}_{t}\right|}{{y}_{t}} +\infty \right\}=\infty$. The MAPE error is not applicable due to its divergence to infinity under the condition of just one sample being zero-valued. At the same time, it is important to note that although it is most often used to evaluate the accuracy of predictive models ^29^, ^31^, REF _Ref119320558 \r \h ^32^ as a kind of error which appears to be scale-independent and it is especially recommended for use in the predictive model evaluation, it actually favors data with low dynamics. Some studies use the maximum value of MAPE as a model evaluation metric (Candelieri et al. ^31^, among others). In order to preserve the possibility of comparing the obtained research results, given the disadvantages of using the MAPE error, the authors in the following section used the ${MAPE}_{max}$ error given by Eq. (5):

5 $$MAPE_{\max } = \frac{1}{n}\sum\nolimits_{t = 1}^{n} {\frac{{\left| {\hat{y}_{t} - y_{t} } \right|}}{{\max (\hat{y}_{t} ,\;y_{t} )}}} \cdot 100\%$$

This modification ensures that the maximal value from a pair of observed and predicted values will be used as a denominator. Usually, this modification prevents the metric from diverging into infinity. However, this modification is based on the assumption that imperfectness of the predicting algorithm provided a forecast error making at least one value from a pair of observed and predicted samples as non-zero one. If model returns prediction equal to the zero-valued observation then the indeterminate form appears in the computation rendering the metric useless $\underset{x\to {0}^{+}}{lim}\underset{y\to {0}^{+}}{lim}\frac{x}{y}=\frac{0}{0}$. Such an occurrence is possible for algorithms depending on the divisions of solution space into subspaces instead of performing linear transformations of the input data, like Decision Tree or K-Nearest Neighbors used as a regressor. Such algorithms may respond with the forecast being equal to zero under the specified input.

Due to the rationale described above, the authors recommend the use of *MAE*_*OM*_ error metrics. This metric provides information on the relative value of error in regards to the mean of the signal. It is both straightforward interpretable like MAE error and ensures independence of the magnitude of water consumption signal from flow volumes. During the numerical experiment water consumption series from different buildings are summed up in order to obtain aggregated signal time series, which in turn increase the signal magnitude with each additional building rendering MAE error incomparable between different cardinalities of data sets aggregated into one time series. Despite that, ${MAE}_{oM}$ metric remains resistant on the increasing magnitude of water consumption series and allows for comparison of following cardinalities of data sets.

### Analysis of variance

The analysis of variance is used in order to establish the minimal amount of water meters, for which predictive error distribution is no longer distinguishable from maximal available amount of data. If predictive error distributions of aggregation of $n$-water meters and maximum number of water meters are not distinguishable, then no significant amount of information is introduced to the system that could help in water consumption forecast, so the aggregation of more water meters does not serve the purpose of making the system more deterministic. The analysis of variance is performed along the Tuckey’s tests in order to establish whether difference in error distributions is significant.

## Case study

**Figure 2:**
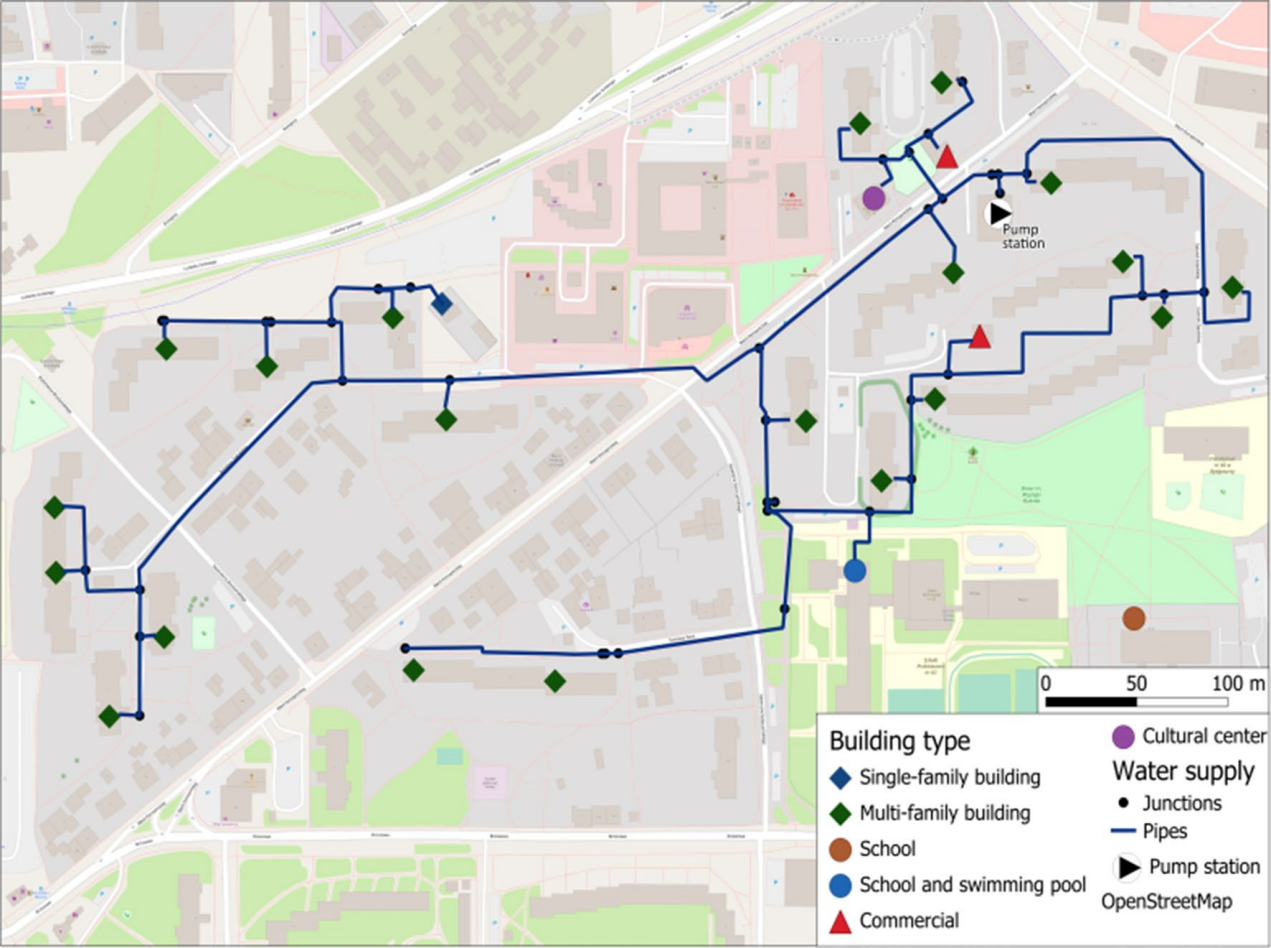
Water supply network diagram showing the researched zoneFor the measurement campaign, telemetry modules were installed on all water meters, which allowed for high-frequency readings. These modules recorded individual pulses from the water meter over time and sent the information via GSM modems to an aggregate database. A dedicated acquisition system automatically recalculated the readings from the water meters and visualized them in the form of time series of temporary flows. These values were then aggregated into 15-min and 1-h series stored in the pooled database. Measurements of water consumption by end users lasted from January 1, 2021 to June 30, 2021. Verified and stored in the database, the six-month time series of hourly water consumption by individual end users provided input for further numerical analyses.

The study used measurement material collected during a measurement campaign carried out on the water distribution network in Bydgoszcz (Poland), which is under the management of the Miejskie Wodociagi i Kanalizacja (Municipal Water Supply and Sewage Company, MWiK). The measurement material consisted mostly of registrations of water consumption by end users located in one of the DMA zones This zone arose from the need to ensure water supply at the appropriate pressure to multi-family buildings, characterized by high buildings in relation to the other facilities in this area of the city. Water is pumped to the analyzed DMA zone by means of a local pump station, which supplies water to a total of twenty-two end users. A diagram of the analyzed DMA zone is presented in Fig. 2.

Sixteen large block-type multi-family buildings, one smaller building that housed a community centre, one single-family house, two commercial-service pavilions and a school are located in the analyzed DMA zone. One of the commercial-service pavilions was permanently closed at the time of the measurements and did not collect water, so it was excluded from further analysis. It should also be noted that out of the surveyed facilities, three multi-family buildings are supplied by two separate connections, making them equipped with two separate water meters. The school consists of two buildings supplied with individual connections, one of which is equipped with an indoor swimming pool and is fed from the DMA zone in question.

The metering campaign additionally covered two facilities, i.e. the second of the school buildings and one multi-family building with two connections, located near the DMA zone under consideration, but already supplied from a different pumping station.

All connections in the facilities covered by the metering campaign are equipped with single jet turbine type water meters with different diameters and measurement resolutions. For water meters with diameters of DN15-20, the resolution is 1 pulse per 1 dm^3^, while for diameters from DN32 and above, the resolution of water meters is 1 pulse per 10 dm^3^.

## Results and discussion

### DMA zone parameters

**Figure 3:**
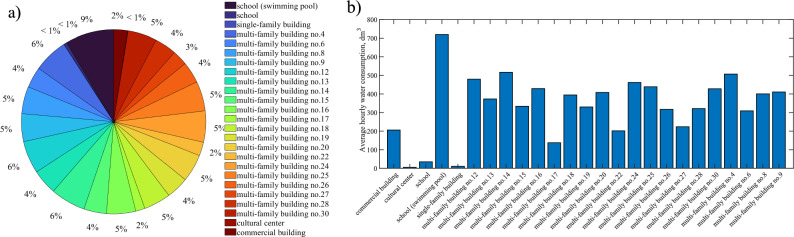
Diagram of water consumption in the selected DMA zone (**a**) and average hourly water consumption in individual buildings (**b**).

A diagram of the water consumption in the selected DMA zone is shown in Fig. 3a. Since the selected DMA zone is mainly dominated by multi-family housing, more than 85% of the injected water is taken for the domestic purposes of its residents. About 9% of the water is supplied to a school that has a swimming pool and 2% to a commercial building. Consumption of less than 1% for the entire zone is observed in a single-family building, a school and a cultural center. Since the study was conducted during the COVID-19 pandemic, the restrictions imposed caused changes in the hourly distribution of water consumption per day in the facilities analyzed. This issue in the case of multi-family housing facilities was the subject of detailed studies^33^. In general, within the DMA zones and the facilities located in them, it is necessary to reckon with the phenomenon not only of temporal variability in water consumption, recognized in the cited work by Dziminska et al.^33^, but also with the aspect of spatial variability^34,35^. As the cited studies showed, spatial variability in water consumption depended mainly on the share of residential, commercial or industrial area in a particular DMA zone. Returning to the analyzed DMA zone in Bydgoszcz, the volume of water consumption within multi-family buildings shows similarity and, on the scale of the entire DMA zone, usually accounts for about 4–5%, although there are buildings with a consumption of only 2% of the injected water. This is influenced by obvious factors such as the size of the building, the number of residents, the standard of apartment furnishings and other sociodemographic factors^16,36^.

A bar graph of average hourly water consumption during the period analyzed is shown in Fig. 3b. The average hourly water consumption for domestic purposes of residents of multi-family buildings in the analyzed DMA zone is 371.2 dm^3^. The customer to whom the largest volume of water is supplied is a school with a swimming pool, with an hourly average of almost 720.0 dm^3^. The cultural center consumed the least amount of water, which was due to the closure of these facilities as a result of restrictions during the COVID-19 pandemic. The same applies to the school, which at the time implemented a distant learning mode.

### Forecasting water consumption for individual buildings

The first stage of the analyses was to forecast water consumption individually for each of the building types in the DMA zone, which is the study area. The goal was to see how large the differences in the obtained prediction errors would be between different building types, where water consumption shows different temporal variability. Predictions of water consumption for individual buildings were made using the Random Forest algorithm, since at this stage (as in the work of Smolak et al.^25^) the smallest considered errors ${MAE}_{oM}$ and ${MAPE}_{max}$ were obtained.

**Figure 4:**
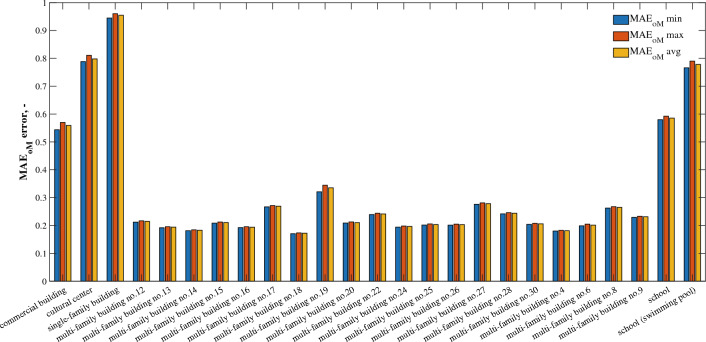
${MAE}_{oM}$ bar diagram for individual types of buildings.

${MAE}_{oM}$ error clearly indicates (Fig. 4) that water consumption forecasting shows the lowest accuracy for facilities such as school, school with swimming pool, commercial building, cultural center and single-family building, where ${MAE}_{oM}$ is in the range of 0.57–0.96. Facilities of this type are characterized by the highest randomness of water use due to the fact that they have relatively fewer users compared to multi-family buildings. Thus, it becomes more difficult to know certain behavioral behaviors in water use^37^. With regard to schools with swimming pools, it should be noted that pool filling periods are also irregular and depend only on the need to replace the water after it loses its quality standards. In addition, in this type of facility, the six-month observation period conducted at the peak of the COVID-19 pandemic proves insufficient for recognizing the determinism of water consumption, so that prediction errors remain high. The ever-changing restrictions associated with the COVID-19 pandemic resulted in the fact that in buildings of a recreational, cultural, educational, service nature, the period of research conducted was not pertinent to the possibility of obtaining representative patterns of water consumption. Moreover, as shown in the research by Benítez et al.^38^, when comparing single-family forecasts to multi-family forecasts, water consumption in single-family housing by each user has an important weight in the signal and thus in the variance, causing a rather irregular time series in which the lack of regular patterns could be observed. This situation was further exacerbated by the COVID-19 pandemic, as shown in the research conducted for the same zone by Dzimińska et al.^33^.

The situation is different for multi-family buildings. The average error of the water consumption forecast, expressed in units of water volume, oscillates between 37 and 110 dm ^3^, which corresponds to error ${MAE}_{oM}$ at the level of 0.17–0.34.

**Figure 5:**
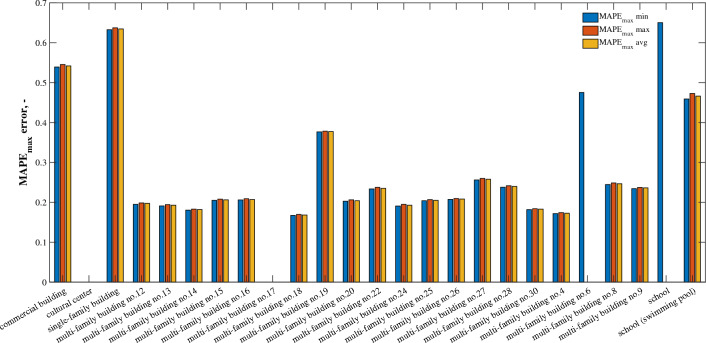
${MAPE}_{max}$ bar diagram for individual types of buildings.

According to the methodology chosen, where the model accuracy was also evaluated using error ${MAPE}_{max}$, Fig. 5 shows the achieved values of this parameter for forecasting water consumption for individual buildings. The results show that for buildings other than multi-family ${MAPE}_{max}$ exceeds 50%, while for multi-family housing it ranges from 17 to 47%. For comparison, the problem of water consumption forecasts for water meter data from individual buildings was addressed by Candelieri et al.^31^. As in the present study, the authors projected water consumption based on AMR data, obtaining a MAPE error from half of the water meters of less than 30%, while there were also predictions with an error close to as much as 100%. With regard to the present study, a threshold of less than 30% was achieved for 16 of the 19 facilities analyzed. Compared to forecasts created for entire DMA zones, where errors of around 10% can be obtained^25^, the results of the study show the difficulty of predicting water consumption for a single building as a result of less determinism in the data. Velasco et al.^30^ and Benítez et al.^38^ predicted water consumption with respect to domestic and industrial area, obtaining clearly better results in the prediction of water consumption for domestic residents than other non-domestic, which is also confirmed by studies conducted in Bydgoszcz.

The impossibility of calculating the metric for facilities such as a cultural center and a multi-family building, among others, is due to the fact that ${MAPE}_{max}$ metric that possesses the mathematical flaw explained in the section describing this metric. These buildings contain periods of lack of water consumption described as a series of zeros. Correct prediction of a series of zeros is another series of zeros, thus the computation of error can be described as $\frac{0}{max(\mathrm{0,0})}=\frac{0}{0}$ giving in result indeterminate form. Such problems are not present during the computations of the ${MAE}_{oM}$ metric (Fig. 4). The Numpy computational library does not have an expression for indeterminate form, so it returns the infinity. For this reason, the minimum ${MAPE}_{max}$, e.g. for building no. 6 and schools, achieve such values. The error may appear in certain experiments while not occurring in other ones due to randomized division of the dataset into train set, validation set and test set. Each building had 30 experiments conducted, so some buildings could have correct measurements computed despite having a zero values at some points of water consumption series, due to this lack not being present in the test set via random selection. The authors decided to keep both empty results and results with only minimal ${MAPE}_{max}$ in order to emphasize the problem with the metric and give an estimate for multi-family building no.6 and school what kind of prediction accuracy can be expected from these buildings.

A correlation analysis was carried out to see if the accuracy of the prediction and thus the model's ability to achieve the deterministic mark is affected by the number of residents and the number of apartments in the building. Spearman's rank correlation coefficient was used. The multi-family buildings under consideration have between 40 and 84 apartments, occupied by between 69 and 168 residents. Significance of correlation coefficients with *p* < 0.05 was obtained only between the forecast error and the number of apartments. For the number of residents, the correlation coefficient with the forecast error was low, i.e. 0.58, and did not show statistical significance, while for the examined relationship the forecast error—number of apartments was 0.73. The lack of an apparent relationship between the number of residents and the forecast error is probably due to the varying model and the number of family members living in each apartment. Thus, an open question remains as to from how many water meters the measurements will be subject to less randomness and the determinism of water consumption will contribute to the possibility of creating reliable water demand patterns, usable not only in hydraulic modeling, but also burst detection^39^.

### Forecasting water consumption for groups of multifamily buildings

Every machine learning algorithm was evaluated on following increasing sets of water meters 30 times. With each iteration of the experiment the new joined set of data from water meters was randomly created from the original set of water consumption measurements acquired during DMA monitoring campaign. The time series representing each water meters were aggregated and then randomly divided into three subsets: train, validation and set. Table 1 presents the results of each model test evaluation on the set containing maximal number of aggreged water meters data (in total originating from 19 objects). Table 1 presents both average, minimum and maximum value of ${MAE}_{oM}$ oraz ${MAPE}_{max}$ obtained from 30 iterations.

**Table 1:** Acquired prediction errors ${MAE}_{oM}$ and ${MAPE}_{max}$ (%) using different types of algorithms.

Algorithm	${\mathrm{MAE}}_{\mathrm{oM}}$ max	${\mathrm{MAE}}_{\mathrm{oM}}$ min	${\mathrm{MAE}}_{\mathrm{oM}}$ avg	${\mathrm{MAPE}}_{\mathrm{max}}$ max	${\mathrm{MAPE}}_{\mathrm{max}}$ min	${\mathrm{MAPE}}_{\mathrm{max}}$ avg
**SVR**	**7.9**	**7.3**	**7.6**	9.6	8.6	9.0
RNN	8.7	7.3	8.0	9.8	8.4	9.0
FC2	9.3	8.2	8.7	11.4	9.5	10.4
Random forest	9.2	8.6	8.9	10.0	9.3	9.6
XGBoost	9.4	8.5	8.9	10.0	9.3	9.6
KNN	11.5	10.8	11.1	11.6	11.0	11.2
Decision tree	12.5	11.0	11.8	13.0	11.2	12.3

Significant are in value [bold].

According to the results presented in Table 2 the SVR model obtained on average the best results while being trained on joined dataset from all 19 objects. ${MAE}_{oM}$ min was 7.3%, while its counterpart for error ${MAPE}_{max}$ was 8.6%. The worst accuracy of predictions was obtained using Decision Tree, for which errors ${MAPE}_{max}$ and ${MAE}_{oM}$ in the worst variant were 13.0%, and 12.5%, respectively. With regard to what was mentioned in the previous section, the forecast error at the level of the final water consumer, i.e. for the terminal water meter due to the burden of high randomness on the data rarely reaches values below 30%. Such a low error value for the SVR algorithm (i.e., 7.3–7.9%) indicates that not only is it possible to develop a reliable model for water consumption forecasts at the level of a single building rather than the entire DMA zone, but, last but not least, an algorithm characterized by an uncomplicated design, fast computation time and simplicity of implementation can be used for this purpose.

**Table 2:** Analysis of variance results.

Algorithm	Number of water meters	${\mathrm{MAE}}_{\mathrm{oM}}$ avg (%)	${\mathrm{MAE}}_{\mathrm{oM}}$ avg with max. number of water meters (%)
Decision tree	13	12.9	11.8
FCNN	14	9.4	8.7
KNN	10	12.1	11.2
Random forest	12	9.7	8.9
RNN	13	8.7	8.1
**SVR**	**11**	**8.9**	**7.6**
XGBoost	11	10.1	8.9

Significant are in value [bold].

**Figure 6:**
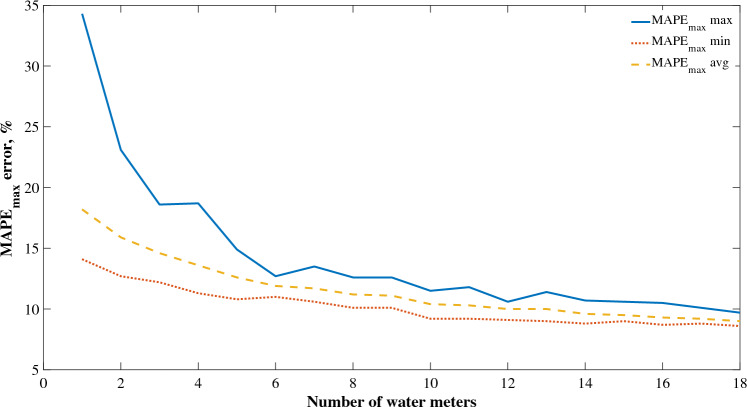
${MAPE}_{max}$ curve for SVR algorithm.

**Figure 7:**
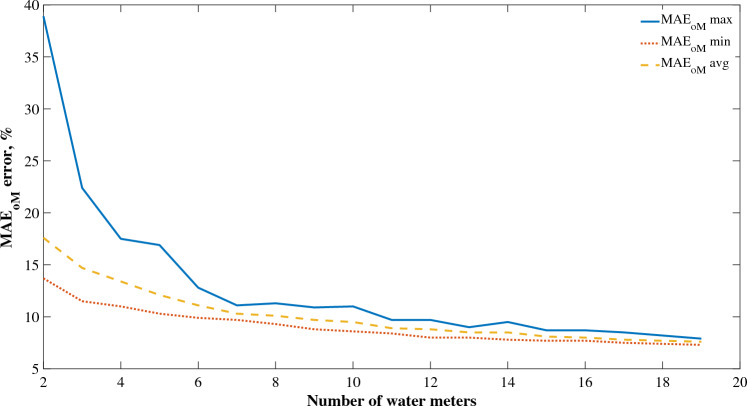
${MAE}_{oM}$ curve for SVR algorithm.

The performance of SVR algorithm, based on ${MAE}_{oM}$ and ${MAPE}_{max}$ on subsequent number of water meters of multi-family buildings from 2 to 19 was presented in Fig. 6 and 7 (for recommended ${MAE}_{oM}$). The error decreases as the number of water meters time series aggregation increases, which is visible for both ${MAE}_{oM}$ and ${MAPE}_{max}$. This means that as the error decreases, the system becomes more deterministic. The average value of ${MAPE}_{max}$ reaches a value of less than 10.0% only at 15 water meters, while for the average value of the ${MAE}_{oM}$ this is already achieved when analyzing 9 measuring devices. The present results indicate the possibility of building reliable models, with a prediction error of less than 10.0%, based on time series determinism for water consumption using 9 water meters simultaneously for a given zone or sub-zone of DMA. However, for this statement to be fully conclusive, it is necessary to apply analysis of variance in the next stage of research.

### Analysis of variance

The determinism of the system rises as the amount of aggregated buildings increases. However, the important question is which number of water meters is indispensable to already deterministic enough system. In order to answer this question the Analysis of Variance (ANOVA) and Tuckey’s tests were conducted. These tests were aimed on finding for each algorithm the smallest number of buildings, which distribution of errors sampled from 30 experiments has been deemed indifferentiable from error distribution for each model obtained on maximum number of buildings. The results are presented in Table 2.

The results depicted in Table 2 suggest that the number of water meters facilitating the deterministic system ranges between 10 and 14. The SVR model, that turned out to be the most accurate in forecasting the water consumption, achieved deterministic saturation on time series created from the aggregation of 11 buildings. Analysis of variance showed that of all the implemented algorithms, the smallest error average ${MAE}_{oM}$ was obtained using SVR. For data including 11 buildings at 8.9% and for data of all buildings at 7.6%. The result obtained is encouraging, bearing in mind that SVR algorithm is one of the least computationally expensive machine learning algorithms. Its inference boils down to perform the inner product of the input vector with its internal weights. It can be said unequivocally that having a model built based on metered data from 11 multi-family residential buildings, the determinism of water consumption becomes apparent and the area in which they are located may constitute a smaller DMA sub-zone for which it becomes cost-effective to install a metered master device.

The very aspect of forecast implementation based on high-frequency water meter data has many advantages. Consumer-level water consumption forecasts, apart from the benefits for water supply companies, such as more effective and efficient water distribution, bring also other benefits for the consumers. They support pro-ecological approach towards water saving by raising consumers’ awareness of water consumption. As shown in the research, such solutions support the idea of Sustainable Cities. Data recording, its visualisation, forecasts along with best practices, water consumption culture and ongoing maintenance can contribute to annual saving of USD 10 633 for a single building ^8^. Moreover, the water consumption forecasts result in faster failure diagnosis, because each increase in forecast error may imply an anomaly^24^.

## Conclusions

The field of data sciences, which is also developing rapidly in terms of the use of analytical tools in utility consumption inference processes, makes it possible to improve forecasts of both the volume of water consumption and the appearance of anomalies in the water distribution system. On the one hand, access to reliable real-time data provides a diagnostic source for water network operators, on the other hand, there are very few literature and implementation reports on the application of high temporal resolution water meters, in relation to individual residential buildings, within which the resulting information and data in excess may constitute streams of data, impossible to interpret in the short term.

The research presented in this article helped to fill the gap and the lack of a clear answer to the question of how many facilities at one time the water meter data can form the basis for building a reliable deterministic model of water consumption. Tests conducted using data from high temporal resolution water meters of a selected DMA zone in Bydgoszcz, where 85% of the water injected into the system is consumed for domestic purposes by residents of multi-family buildings, showed that the most reliable predictions for individual buildings were obtained for multi-family housing (${MAE}_{oM}$ at the level of 0.17 – 0.33). While the number of residents alone showed no correlation with the achieved prediction errors, tests of significance of the Spearman correlation coefficient unequivocally showed the existence of a statistically significant relationship between the number of dwellings and the accuracy of the prediction. For service buildings, a school or a community center, the predictions were at a much worse level, but what is worth remembering is that the prediction of water consumption burdened with high randomness in buildings of this type and industrial plants should be carried out on the basis of individual, dedicated models.

The results show that it is possible to build reliable water consumption models with a prediction error of less than 10.0% for water meters of multi-family buildings. ANOVA analysis showed that with respect to the results obtained for the prediction of water consumption in a set of multi-family buildings using SVR (as the best-accuracy model, with ${MAE}_{oM} avg$ amounting to 8.9%), water consumption determinism was achieved when using data from at least 11 buildings. The study showed that the SVR algorithm, simple in design, easy to implement and capable of performing calculations in a short period of time, allows for smaller prediction errors than advanced methods such as XGBoost or RNN.

The study authors recommend using the ${MAE}_{oM}$ metric to evaluate the accuracy of the water consumption model. The nature of the water consumption data means that evaluation via the MAPE error, which is standard in the literature, results in over-interpretability of the results obtained. The MAPE error is not applicable due to its divergence to infinity under the condition of just one sample being zero-valued. Although it is most often used to assess the accuracy of predictive models as a kind of error which seems scale-independent and it is especially recommended for use in the predictive model evaluation, it favors data with low dynamics.

In future studies, a continuation of the research undertaken, the authors plan to focus on the possibility of anomaly detection through predictive models of water consumption, based on data obtained from high temporal resolution water meters using computational intelligence.

## Data Availability

For data sources, see the Acknowledgments section; on analyses in this manuscript, please contact: justyna.stanczyk@upwr.edu.pl.
